# Virtual Reality for the Prevention and Cessation of Nicotine Vaping in Youths: Protocol for a Randomized Controlled Trial

**DOI:** 10.2196/71961

**Published:** 2025-05-15

**Authors:** Belinda Borrelli, Daniel Weinstein, Romano Endrighi, Nikki Ling, Kathleen Koval, Lisa M Quintiliani, Kaitlyn Konieczny

**Affiliations:** 1 Center for Behavioral Science Research Boston University, Henry M. Goldman School of Dental Medicine Boston, MA United States; 2 Tufts Medical Center School of Medicine, Tufts University Boston, MA United States

**Keywords:** vaping, teens, virtual reality, prevention, nicotine, coping, mood management, depression

## Abstract

**Background:**

Only a few trials target high school-aged teens with vaping interventions, typically focusing on prevention rather than cessation, with content limited to vaping harms and refusal skills. Given the co-occurrence and increasing incidence of vaping and mental health issues in teens, both must be simultaneously addressed by vaping interventions.

**Objective:**

This randomized controlled trial aimed to evaluate the feasibility and preliminary efficacy of a virtual reality (VR) intervention for teens that targets both determinants of vaping (eg, mood and stress) and vaping prevention and cessation skills.

**Methods:**

The participants are 150 students attending 1 of 2 racially and ethnically diverse Boston-area high schools. Health classes are randomly assigned to VR or control (assessment only). Students are eligible if not opted out by their parents, provided assent, and did not have health issues that preclude participation. While control classes (and ineligible students within them) have their regular classroom activity, VR classes are provided with Oculus Meta Quest 2 headsets and engage with the intervention during 3 class periods, once per week. Players solve a mystery with a vaping subplot and play a series of minigames to teach emotional regulation, coping strategies, and building resilience. A mobile app connects to the VR game, providing a home-based experience to reinforce skills learned. VR and control classes complete baseline and postintervention surveys on the same schedule. Primary outcome measures include feasibility, satisfaction and engagement with game play, and vaping awareness, knowledge, attitudes, and intentions to avoid or quit vaping. Secondary outcomes include vaping quit attempts, 7- and 30-day point prevalence abstinence, motivation and self-efficacy to quit or avoid vaping, positive and negative affect, emotional regulation and coping, and motion sickness.

**Results:**

The project was approved by the institutional review board on April 2, 2024, and data collection began in April 2024 and concluded in June 2024, and 150 participants were recruited (98 intervention and 52 control). Although freshmen and sophomore classes were targeted in this study, there were several juniors and seniors (n=5) in these classes due to school transfers and the need for them to take the mandatory curriculum. Data cleaning has been completed, and analyses are ongoing. The results are expected to be submitted for publication in August of 2025.

**Conclusions:**

As VR is visual, auditory, and tactile, it is ideal for practicing skills, facilitating memory, and increasing the likelihood of transfer to real-world settings. The immersion of VR promotes engagement with the intervention, rather than external digital temptations. Our study is innovative due to the cluster-randomized design, focus on both prevention and cessation, integration of mood management, and implementation among racially and ethnically diverse teens. Incorporation into school-based health curricula allows for proactive reach.

**Trial Registration:**

ClinicalTrials.gov NCT06003439; https://clinicaltrials.gov/study/NCT06003439

**International Registered Report Identifier (IRRID):**

DERR1-10.2196/71961

## Introduction

The prevalence of e-cigarette or vaping product use among teenagers is a substantial concern, with 10% of middle and high school students in the United States self-reporting current e-cigarette use in 2023, which is approximately 2.8 million students [1]. Moreover, 34.7% of current e-cigarette users report use on at least 20 of the past 30 days [1]. Though many teens believe e-cigarettes are a safer alternative to combustible cigarettes [2], studies suggest e-cigarette use has negative physiological, cognitive, and behavioral effects for teenagers [3]. E-cigarette use is associated with a variety of mental health issues, including depression, internalizing problems, perceived stress, and suicidality [4-6]. Moreover, the percentage of high school students experiencing persistent feelings of sadness or hopelessness (for at least 2 consecutive weeks) increased from 28% in 2011 to 42% in 2021 [7,8]. Therefore, e-cigarette interventions should include emotional regulation and coping in addition to typical approaches that involve teaching vaping refusal skills and managing cravings.

Several intervention approaches have been developed and tested with older adolescents, such as text messaging [9], contingency management [10], and social media approaches [11], but few studies have tested vaping interventions for high school-aged teens [12]. One trial among high school-age teens randomly assigned participants to watch Food and Drug Administration “Real Cost” advertisements (on either health harms of vaping or addiction potential) or a control group (neutral videos about vaping) with results showing that both intervention groups had lower self-reported likelihood of future vaping, less positive attitudes toward vaping and vaped fewer days per week compared to those in the control group [13]. In a small pilot study (n=69), text messages regarding the harms of e-cigarettes were sent to teens for 6 days. Outcomes were measured on day 7, with participants reporting increased knowledge and worry regarding the risks of e-cigarette use [14].

Another intervention approach used with high school-aged children is virtual reality (VR). VR is an immersive, computer-generated simulation that enables users to navigate within a 3D environment and interact with objects within the environment. One study used a pre-post design to test a VR program that focused on teaching vaping refusal skills (n=47) during 2 play sessions [15]. The results demonstrated a significant increase in knowledge and perceptions of e-cigarette harm, and a significant decrease in perceived likelihood of using e-cigarettes in the future, but there were no significant changes in attitudes toward e-cigarettes, social norm perceptions, or refusal self-efficacy. However, this study was not randomized, and 38 of the 47 study participants were boys. A follow-up study that randomized students by class tested the efficacy of the program in 285 middle school students aged 11 to 14 years [16]. The results showed that the VR program significantly increased e-cigarette knowledge and perceived addictiveness of e-cigarette at the 6-month follow-up, but there were no differences in e-cigarette behaviors between the control group and the VR group [16].

There are several advantages to VR interventions. VR is a 360-degree experience, so distractions by external stimuli are limited (eg, temptations to check social media). The immersive nature of VR also promotes learning and behavior change by increasing attention [17] and by creating meaningful emotions and experiences [18]. In VR, skills are not only learned auditorily and visually but also through tactile interaction. These multiple modes of skill acquisition increase memory retention. Finally, VR is an engaging medium for teens [16].

We describe the protocol for a randomized controlled trial that evaluates the feasibility, engagement, satisfaction, and acceptability of a VR program aimed at the prevention and cessation of vaping among high schoolers, delivered as part of their regular health curriculum throughout 3 classes (Clinical Trial Registration NCT06003439). A secondary aim is to assess the preliminary impact of the intervention on the determinants of vaping and vaping behavior. Distinct from the prior VR studies that focused solely on refusal skills for vaping [15], the intervention developed for this study focuses on the determinants of vaping (emotional regulation, coping strategies, and resilience) in addition to prevention and cessation skills (refusal skills, craving management, and cessation resources). A mobile app was also developed for use by students at home to reinforce the skills learned during the VR experience and to continue interactions with game characters in between VR sessions. Our study also expands upon prior literature by including a randomized controlled trial design and by including an ethnically and racially diverse sample. Our overall aim was to test the feasibility, acceptability, and preliminary efficacy of a VR-based prevention and cessation intervention program for high school-age students. We hypothesize that the intervention will be feasible (eg, high rate of student assent), satisfactory to students, and that most students will engage with the intervention (eg, complete all 3 sessions). Although the trial is not fully powered, we further hypothesize that the intervention will have promising effects in the desired direction on vaping attitudes and behaviors.

## Methods

### Participants

Eligible participants are assenting students enrolled at 1 of 2 high schools in the Greater Boston Area of Massachusetts. Exclusion criteria are (1) prone to seizures or severe motion sickness; (2) visual impairments that would prevent them from playing VR or using the VR device; (3) parent or guardian opt out; and (4) indication by school staff that participation is inappropriate (eg, heart condition, visual or auditory disability, psychiatric disorder, pacemaker, hearing aid, implanted medical device, or any other medical reasons).

Opt-out forms were distributed to eligible students’ parents or guardians that detailed this study’s procedures, potential risks, as well as how to opt their child out of this study. The opt-out form was translated into languages spoken by the families of the children who attended the schools and distributed approximately 3 weeks before obtaining assent from students. Parents or guardians were able to contact either the school or research staff to opt out their child. During their health class, electronic assent forms are given to all students who have not been opted out. Human subjects approval was obtained from our institutional review board (H-43102), and subsequent protocol modifications were submitted to the institutional review board. After assent, students complete a baseline survey, and data are stored in REDCap (Research Electronic Data Capture; Vanderbilt University), a secure, web-based app designed to support data capture and features to support cleaning, storage, and analysis.

### Study Design and Random Assignment

Health education classes in the 2 high schools were randomized to either intervention (VR) or control (assessment only) by a statistician external to this study, using a 1:3 threshold probability. Three classes were randomized in 1 high school, and 4 classes in the other high school. Regarding the latter high school, 2 classes were “yoked” during randomization due to small class sizes. After completing a baseline survey, classes randomized to VR engage with the VR intervention once per week for 3-4 weeks, followed by a follow-up survey. Classes randomized to control complete baseline and follow-up surveys only, on the same schedule as the VR group. All study procedures take place during regular health classes. Students in control classes, as well as ineligible and nonassenting students in the VR classes, engage in alternative assignments regarding vaping or substance use from their respective teachers while study procedures are conducted. Only eligible participants who assented to be in this study completed pre- and post-assessments. As randomization occurs at the class level, teachers cannot be masked to the condition. Due to the nature of the intervention, study staff and participants were also not masked to the condition.

### Equipment

Participants in the VR condition are provided with Meta Quest 2 VR headsets, which do not need to be tethered to computers. Each headset has adjustable head straps and lens spacing, and 3D positional audio. The headset is equipped with cameras to track the movement of the head and body and read hand gestures, so that hand controllers are not needed for navigation through the digital world, to execute game play, or to interact with the digital environment (eg, grabbing or throwing objects). Head straps and the facial interfaces are disinfected with antibacterial wipes after each use.

### VR Intervention

#### Overview

Our prior mixed methods studies informed the structure and content of the intervention, in which we conducted focus groups and surveys to identify: teens’ intrinsic and extrinsic motivators to initiate and maintain vaping; the methods teens currently use to quit vaping and perception of extant vaping cessation treatments; and intervention delivery methods and components that are acceptable to teens [19,20]. We also conducted usability testing with teens that consisted of playing progressive iterations of the VR program and interviewing them about their play experiences. This helped to identify bugs, ensure functionality, and make content changes.

#### VR Play Session Procedure

There are 3 VR play sessions, and each is 30-40 minutes in duration (approximately 1 class period) and played while seated. Four class periods are reserved for game play so that absent students can “make up” missed sessions. The VR sessions occur in large areas that allow for entire classes of students to use the VR headsets simultaneously (ie, school gymnasium or open space in library) without injury (eg, chairs that are not attached to desks).

Students download the study’s mobile app onto their personal devices to obtain a login for the VR program and also to engage with game content at home. Students who do not have compatible devices can be logged into the VR game via study devices. Students are assisted with donning the VR headsets, including adjustment of the head straps and the interpupillary distance of the lenses, and providing spacers for eyeglasses, if needed. Students are given a brief overview of VR, including how to navigate through a digital environment (eg, teleporting), how to interact with objects in the environment (eg, grab, place, and throw objects), how to interact with characters in the environment (eg, waving to initiate communication with characters), and an overview of the minigames.

#### VR Play Sessions

**Figure 1:**
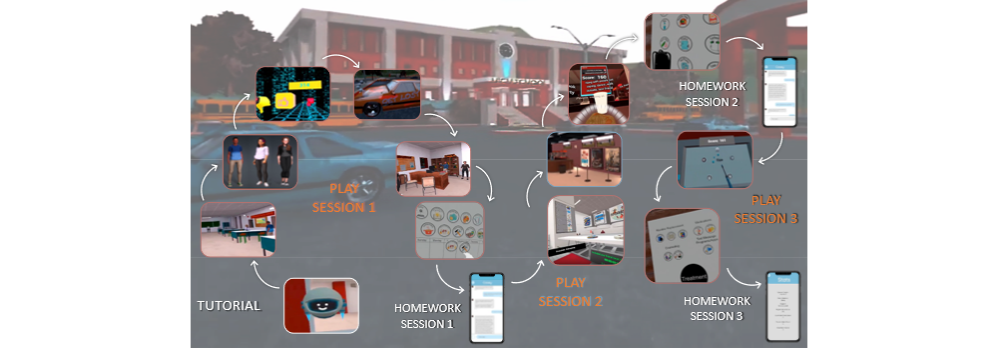
Player journey in the VR intervention. VR: virtual reality.

The 3 play sessions are connected by a narrative arc that has a main storyline (the player solves a mystery at a fictional high school) and a vaping subplot. During the vaping subplot, the player chooses a friend at the high school, and the player discovers that the friend has a history of being caught vaping at school. During the story, the friend experiences triggers to vape, struggles with the decision of whether or not to quit vaping, tries to quit unsuccessfully, and finally applies a constellation of coping and motivational strategies to successfully quit. The player tries to help his or her friend quit vaping through various means, including playing 5 minigames to learn about how to cope with negative moods, reduce the friend’s craving to vape, and identify treatments for vaping cessation. The minigames also go beyond vaping to teach general coping skills and building resilience, so that these skills have broad applicability to a wide range of risk behaviors and mood problems. At the end of each play session, the player and friend go to a secret cabin in the woods behind the high school, which has an “art wall” with additional intervention strategies for at-home practice. A friendly robot, ARAL, guides players through the overall game. A mobile app is connected to the game, where players can view their scores and send text messages with their in-game friend, providing a home-based experience (outside of class time) to reinforce skills learned in the classroom-based VR game. No new intervention strategies are presented in the mobile app; rather, the text message conversations relate to the events occurring in the VR game, as well as topics discussed with their fictional friend during game play (eg, the friend fighting urges to vape and what coping skills they used). Due to technical issues, actual use of the mobile app was not measured. Figure 1 depicts the player’s journey throughout the intervention.

This image depicts the player’s journey throughout the intervention. The VR app is divided into 3 play sessions. Each session ends at an “art wall” that reviews the content learned in that session and how to bring coping skills into the “real world.” Between VR sessions, there is a home-based component where the player can interact with a mobile app that is connected to the game, so they can see their scores and interact with their friend from the game, reinforcing the skills learned in the game. The first VR session introduces the player to ARAL the robot, has the player select a friend and play the first minigame together, and introduces the main plot (the mystery) and the subplot (the friend uses vapes). In the second VR play session, the player and friend try to solve the mystery and interview suspects in different locations (school cafeteria and movie theater), and 2 more minigames are played to further develop coping skills and build resilience. Play session 3 delivers content via 2 more minigames, the players solve the mystery, and also engage in an art wall activity that involves the discovery of different options for vaping cessation.

#### VR Intervention Content: VR Minigames and Art Wall Experiences

##### Rhythm Game

The player learns that physical activity helps reduce stress and cravings to vape. The objective is to hit boxes that fly toward the player to the beat of high-energy music while making the correct gesture (thumbs up, thumbs down, or OK symbol) with the correct hand, with gameplay becoming progressively more difficult throughout 3 rounds.

##### Lunch Line Game

The player identifies ways to cope with cravings to vape and also helps their friend reduce their cravings to vape (higher points lead to lower cravings on their friend’s craving meter). The player grabs the correct coping strategy from the cafeteria shelf to destroy different cravings as they appear on lunch trays that are continuously moving with increasing speed down the lunch line conveyor belt.

##### Popcorn Game

The player identifies their own triggers (for vaping, and for negative moods and stress) and learns coping skills (eg, refusal skills, options for quitting vaping, and stress management). While the player and friend are in a movie theater to search for a suspect, ARAL shrinks them down into a popcorn machine. Kernels pop, and each one is labeled with a trigger. The player grabs each kernel and throws it into 1 of 2 popcorn buckets labeled “applies to me” or “does not apply to me.” In the next round, 3 popcorn buckets appear, each labeled with a coping strategy, and the player must throw the kernel with the trigger on it into the bucket with the correct coping strategy before the “shot clock” runs out.

##### Vision Board Game

Players identify their personal goals (how they envision their future selves) by grabbing and placing them on a vision board, and then they learn how vaping may hinder those goals because a vape cloud appears and moves around the board with increasing speed, attempting to remove the goals. Players must use a spray hose to remove the vape cloud before it erases their goals, and then they must grab and quickly replace the goals that have been destroyed.

##### Art Walls

Intervention strategies include Behavioral Activation [21] (planning and scheduling activities, even if you feel sad), developing a crisis survival kit [22], engaging in a deep breathing exercise with ARAL, and learning about treatment options for vaping. Intervention strategies are delivered by having the player interact with objects (eg, grabbing and placing) and practice them in real time (eg, deep breathing).

#### Control Classes

These classes complete baseline assessments and post-assessments on the same schedule as the classes randomized to VR, but do not receive the VR intervention or vaping prevention or cessation. Instead, they receive their regular health education curriculum from their teachers, which includes required reading and in-class assignments regarding general substance use, and vaping is included as a topic.

#### Measures

Measures are administered via REDCap during class time, using Chromebooks (Google LLC). The follow-up measure is give to both groups approximately 1 week after the VR group completes their final session of the game.

#### Primary Outcome Measures

Feasibility is assessed as the percent of students who assented, the percent who withdrew from playing the VR game, and the percent who withdrew from this study. Engagement is objectively assessed via the VR headset and includes the number of participants who complete the VR game, the amount of time that the game is played by each participant, and the number of game chapters completed (1-10). Satisfaction is assessed with several self-report measures: (1) an overall star rating of the VR program (1-5 stars); (2) 7-item scale of gameplay experience (eg, “I liked the way the game looked” and “I felt connected to my character in the game”), each item rated on a 4-point scale (1=strongly disagree to 4=strongly agree) [15]; and (3) after each VR game session, participants rate the degree to which they liked each play session and each minigame within that play session on a 4-point scale (“I liked it a lot,” “I liked it,” “meh,” and “thumbs down”). An adapted measure of perceived program impact is used to assess how much participants agree (1=strongly disagree to 5=strongly agree) with a series of statements regarding the effects of the program on their vaping (awareness, knowledge, attitudes, behavior, intention to change, help-seeking, and ability to resist peer pressure), and ability to cope with stress, improve their mood, and avoid use of alcohol and other substances [23].

#### Secondary Outcome Measures

Vaping-related behaviors are assessed, including attempts to quit vaping since starting the VR program, use of quit vaping methods (behavioral, pharmacological, and digital), 7- and 30-day point-prevalence abstinence, and frequency of current vaping (not at all, some days, most days, and every day). Motivation to quit vaping (1-10 scale; 1=not at all to 10=very much) and intentions to quit vaping (eg, intention to stop in the next 30 days, 6 months, more than 6 months, and no intentions of stopping) are also assessed [24], as well as motivation to use evidence-based vaping cessation resources (“How much do you want to use at least one recommended treatment for stopping vaping [such as nicotine patches, nicotine gum, nicotine lozenges or counseling?”]) [19,25].

Vaping prevention is assessed with 2 items regarding the degree of motivation and confidence to avoid vaping (1-10 scales; 1=not at all to 10=very much), 1 item on intentions to try vaping (eg, I intend to vape within the next 30 days; 6 months; I may vape at some point in the future but not in the next 6 months) [26], 6 items on self-efficacy to resist vaping across a variety of high-risk situations (eg, “You feel agitated or tense. Are you confident you will not vape”: definitely, maybe, neutral, maybe not, and definitely not”) [27], and 7 items assessing refusal skills and perceived enjoyability of vaping (“If one of your friends were to offer you a vape, how easy would it be to say no to your friend?”; 4-point scale 1=very easy, 4=very hard) [26].

Determinants of vaping are also measured. Coping is measured with the 16-item Adolescent-Coping Orientation for Problem Experiences scale (eg, “When you face difficulties, how often do you...try to think of the good things in your life”: never, hardly ever, sometimes, often, most of the time) [28,29]. Resilience is measured with 6 items regarding the frequency of different situations (eg, small problems got me very upset: never, a little, sometimes, or often) [30]. Positive affect is assessed with the 8-item PROMIS (Patient Reported Outcome Measurement Information System) Pediatric Positive Affect Scale [31], and negative affect is assessed with the 10-item negative affect scale from the Positive and Negative Affect Scale for Children [32].

Tolerability of the VR game is assessed with the 9-item Virtual Reality Sickness Questionnaire [33], which measures the degree to which participants are affected by a variety of symptoms (eg, eyestrain, headache, fatigue, or dizziness) while playing the VR game.

### Data Management

Participants’ data are kept confidential according to our institutional regulations. Participants are identified by ID numbers, with all data stored and managed in REDCap in a secure, HIPAA (Health Insurance Portability and Accountability Act)-compliant server. Unanticipated problems and adverse events are recorded on electronic case report forms and reported to our institutional review board per regulations. As the study is no more than minimal risk, a data safety and monitoring board was deemed unnecessary.

### Sample Size

As this is a pilot feasibility study, a formal power calculation was not conducted. The sample size was determined based on the need to include a sufficient number of students exposed to the intervention, the availability of students in health classes at the participating public schools, and the timeframe allowed by school administrators. This approach allows us to assess our primary outcomes (eg, feasibility, acceptability, engagement, and satisfaction), as well as preliminary impact on relevant vaping-related constructs. We planned on including approximately 80 students in intervention classes and 40 students in control classes. This is based on an average of 25-35 students per class. We conservatively estimated that approximately 15%-20% of students will either not provide assent to be in this study or drop out post-assent. Therefore, we aimed to enroll approximately 150 participants.

### Analytic Plan

Data will be inspected for outliers, and descriptive analyses of baseline differences in sociodemographic and vaping-related characteristics between the VR group and the control group will be conducted using *t*-tests or chi-square tests, as applicable. We will examine differences in key baseline characteristics (eg, gender distribution between groups) so that they may be covaried in analyses. Feasibility, acceptability, engagement, and satisfaction (primary outcomes) in the VR group will be summarized as means and SDs for continuous variables, or number and proportion of participants for categorical variables, as appropriate. We will then report the number and proportion of participants who progressed through each of the 3 play sessions (chapters 1-10) of the VR game. Lastly, game ratings obtained after each play session will be reported as number and proportion of participants rating each category (“I liked it a lot,” “I liked it,” “meh,” or “thumbs down”).

Data on secondary outcomes will be summarized as means and SDs for continuous variables, or number and proportion of participants for categorical variables as appropriate, and compared between the VR group and the control group (baseline and follow-up). If low cell sizes occur, data might be aggregated to support statistical stability in the models. Differences in secondary outcomes between the groups will be evaluated by computing effect sizes (eg, Cohen *d* or odds ratio) as appropriate.

We will conduct analyses on treatment completers (the subsample of participants who completed the entire VR game) as well as intention-to-treat (as randomized). Additionally, we will perform analyses on the entire sample (English learners and English proficient students) as well as conduct analyses separately by English proficiency status. This will allow us to gain invaluable knowledge to inform future research regarding the feasibility of gamified health interventions for students with different levels of English proficiency.

### Ethical Considerations

The Boston University institutional review board approved the project on April 2, 2024 (IRB# 43102). Assent will be obtained from participants via REDCap, and parents will be sent an opt-out form, as per school protocol. Participants did not receive compensation for participation. All data will be deidentified, kept confidential, and stored in a secure location on an institutional server, which is backed up daily. Only certain members of this study’s staff or others designated by the principal investigator will have access to this study’s data. Protocol modifications will be reviewed and approved by our institutional review board and communicated to the American Heart Association.

## Results

The project institutional review board approved on April 2, 2024, and data collection began in April 2024 and ended in June 2024, and 150 participants were recruited (98 intervention and 52 control). Although freshmen and sophomore classes were targeted in this study, there were several juniors and seniors (n=5) in these classes due to school transfers and the need for them to take the mandatory curriculum. Data cleaning and analysis are underway, and results are expected to be submitted for publication in August 2025.

## Discussion

Both vaping and mental health issues are on the rise among teens. Given the relationship between vaping and mental health, both must be simultaneously addressed by vaping prevention and cessation interventions. We developed a VR intervention that focuses not only on the skills needed for the prevention and cessation of vaping but also on skills that are needed to cope with common teen mental health issues, such as stress, negative moods, and building resilience. Focusing only on vaping without attention to mental health is not in line with what teens want in a vaping intervention [19,20,34] and ignores critical determinants of vaping behavior [35,36]. Moreover, our VR app is transdiagnostic, in that vaping prevention and cessation skills can be transferred to other risky behaviors, and the general coping skills can be applied to several mental health issues, including depressed mood and anxiety.

The choice of using VR to deliver the intervention was driven by several factors. First, as VR technology has improved in both graphics and functionality, it is emerging as an engaging and effective method to treat a variety of mental health [37,38] and physical health issues [39,40]. Second, VR allows for visual and auditory intervention delivery, and interactivity with simulated objects and people. This multisensory delivery is ideal for practicing skills, supporting visual and tactile memory, and increasing the likelihood of transfer to the real world. In our study, we took the additional step of developing a companion mobile app that allowed for continued interaction with the characters from the game between VR sessions (while at home) to further reinforce the skills learned in the VR sessions. Third, data has shown that teens enjoy games and VR [15,16,41], making it an acceptable platform for intervention. Fourth, given the “cool” factor of many of the vaping device designs and functions, interventions are needed that are equally intriguing to teens. Finally, the immersive nature of VR allows teens to focus exclusively on the intervention, instead of checking their phones, scrolling social media, or engaging with other digital temptations.

Our study is innovative because it randomizes high schoolers at the class level, targets both prevention and cessation of vaping in teens, integrates mental health coping skills into a vaping prevention and cessation program, and tests VR in racially and ethnically diverse teens. The school-based setting and integration into the regular health curriculum allow for proactive reach to teens, with minimal burden on the school. As this is a pilot feasibility study, the conclusions will be limited given the small sample size and lack of power to detect effects. Additionally, our control group does not control for attention. Using an attention control in VR is costly, requiring additional development or an off-the-shelf product that would sufficiently match all of the user mechanics (eg, throwing, grabbing, etc) to ensure a true matched-control. For example, if effective, initial integration into schools would be a financial investment. However, the price of VR headsets continues to decline. Moreover, 10 years ago, there were no Chromebooks integrated into classrooms, and now every student has one. Another limitation to VR is that students with visual or auditory disabilities would not be able to fully experience VR, and, as with other learning platforms, accommodations would need to be made for these students. However, because VR has visual, auditory, and tactile learning components, it may be easier for English learners to learn concepts and skills through VR, rather than written material. Another advantage of VR is remote learning. If students need extra support for a particular lesson, they could take the VR headset home as homework, or if there is another pandemic, VR could be a platform for remote learning, particularly with a multiplayer platform (other teachers and students could interact in the same simulated environment).

Importantly, our VR program focuses on both prevention among teens who do not currently vape and quitting among teens who do vape. Therefore, it can be broadly applied in school settings to the general student population and not solely used among those who were “caught” vaping. Ideally, the VR program would be used in conjunction with other school-wide and community-wide policies that discourage vaping for maximum effect on reduction in teenage vaping and associated health outcomes.

## Abbreviations

HIPAA: Health Insurance Portability and Accountability Act
PROMIS: Patient Reported Outcome Measurement Information System
REDCap: Research Electronic Data Capture
VR: virtual reality

## Data Availability

The datasets generated and analyzed during this study will be available from the corresponding author on reasonable request.
